# The Hidden Work of Urology Residents - A Cross-Sectional Study

**DOI:** 10.7759/cureus.10668

**Published:** 2020-09-26

**Authors:** Zehra Kazmi, Kaleem Khan, M Hammad Ather

**Affiliations:** 1 Section of Urology, Department of Surgery, Aga Khan University, Karachi, PAK

**Keywords:** urology consults, referrals, consultation, e-consult, urology department

## Abstract

Background: In a tertiary care hospital that caters to all kinds of patients in the clinical and emergency setting, consultation is an important service provided by the urology team. Profiling the spectrum of urologic disease encountered by trainees will assist in the planning of residency curricula and is bound to improve patient outcome for procedural education.

Methods: All urologic consultation requests received over a period of three months (November 22, 2019, to February 22, 2020) were identified and recorded in a prospectively maintained consult log. Information collected for each encounter included the time, date, reason for consult, primary service and diagnosis along with the final urologic diagnosis, any urologic intervention, and basic patient demographics (gender and age).

Results: Over three months, a total of 568 consult requests were reviewed. Of the patients consulted for, 74% were males; the mean age was 58.45 years (SD+/-19.5 years). The most common service seeking urology consult was the Emergency Room (n=240, 42.25%). The most common reason for consultation was hematuria (n=103, 18.13%) followed by obstructive uropathy (n=98, 17.25%). The majority (n=147, 26%) of the calls were placed between mid-day and 4 pm. Of the total, 26% required immediate attention. Urologic intervention was required in 226 (39.8%). The number of consults seen by junior team members was 478 (84.14%).

Conclusion: Hematuria and obstructive uropathy are the most common reasons for urologic consultation requests. Nearly two-thirds of the consults either required immediate attention or intervention. Most of the consults were seen by junior residents, who required elaborate training to address these common issues independently. We believe that our results will be helpful in developing a curriculum for training junior residents.

## Introduction

Clinical consultation requests to a specialty on-call team are an important component of large multi-specialty hospital practice. Much of the literature comes from specialties such as dermatology and palliative care [1]. Data regarding consultation requests to urology is limited and often involves an investigation of consultations for iatrogenic catheter injuries, where authors also highlight the inadequacy of an intern's training to handle such emergencies [2].

Urology consults can broadly be classified into those requiring immediate attention (testicular torsion, priapism, trauma, Fournier’s gangrene, abdominal injuries) or the ones that can be seen within a few hours (mild hematuria, renal colic, prostatomegaly, urinary tract infection with anatomic abnormalities of the urinary tract). Adequate profiling and “triage” of urologic consults as encountered by the on-call residents can help in the development of a standardized curriculum to improve patient outcomes and junior staff member training. The type of consultation request may differ depending upon the patient population and the primary service requesting for urology to be on board [3].

Urology residents at our center spend a greater part of their first year dedicated to the consult services. With this study we aim to identify types of clinical scenarios encountered by the urology team at a tertiary care referral center in order to aid in the continuous development of the resident educational curriculum and to guide resource allocation (staffing, supply stocks, equipment, and ancillary services) to maximize efficiency.

## Materials and methods

Study design

After approval from the Ethical Review Committee, a cross-sectional study was conducted in the Urology Department of a University Hospital. All urologic consultation requests (routine and emergency) were prospectively tracked and logged over a period of three months (November 22, 2019, to February 22, 2020). Data was collected via non-probability consecutive sampling.

Information collected for each consult request included the time, date, reason for consult, primary service and diagnosis along with the final urologic diagnosis, any urologic intervention, and basic patient demographics (gender and age). Patient records with incomplete information were excluded from the study. To protect the identity of patients, all medical record numbers were assigned a study code, and the original data was accessible only by the Primary Investigator.

Urgent encounters were defined as the ones requiring attention within 30 minutes of the request based on the patient's presentation and also at the discretion of the primary team. Routine encounters required urologic assessment within 24 hours. 

Data collection

At our center, all urologic consultations are received on a cell phone assigned to our service, which is linked to easily accessible software. All data was retrieved on a daily basis from the online records. Subsequently, all information was checked on our online system for consultation requests, where it is mandatory for every specialty to document the findings and management, and “close” the request. There was no interaction with any patients throughout the course of data collection or review.

Consult workflow

A urology call team comprises one senior resident (postgraduate year [PGY] V or VI) and one junior resident (PGY I to IV). Although on-call for the same amount of time, it is usually the junior residents who are fielding and evaluating more consults compared to the senior residents. Once the documentation of the encounter is completed by the resident, all information is entered in an easily accessible online application, consolidating all patient-specific records in one place. It is an easily accessible, user-friendly application, and with a stable internet connection, can be used equally well via smartphones and computers. All consults are evaluated by faculty either the same day, or within a 24 hour period, depending on the urgency level required.

The consult is assigned to the primary urologist of the patient (if applicable and available), or to the on-call attending. All consults are also discussed with faculty members prior to finalizing the management plan.

Data analysis

Data analysis was performed using Statistical Package for the Social Sciences (SPSS®) v26 (IBM Corp, Armonk, NY, USA). Descriptive statistics have been reported as frequency and percentages for categorical variables (such as frequency of consultations and the various clinical scenarios in which they were encountered). For quantitative variables (age) mean ± SD/median (interquartile range [IQR]) has been reported as appropriate. Data were also stratified based on age, gender, and whether the consult required any kind of urologic intervention or not.

## Results

Urology on-call cover received a total of 538 consults over three months. Of these, 74% required urgent input, whereas 26% were classified as routine encounters. The average number of consults seen per day was six. Of the total, 418 (74%) were male patients, while 150 (26%) were females (Table 1). Mean age was recorded at 58.5 years (SD+/-19.5 years), with the maximum number of consults received for patients aged 65 years and above (n=238). For the purpose of this study, the day was divided into six four-hour periods. The highest number of consults were received from 12 pm to 4 pm. The majority of the consults were generated by Emergency Medicine (n=240, 42.25%), followed by Internal Medicine (n=158, 27.81%). Urologic consultation requests from various other services are mentioned in Table 1.

**Table 1:** Demographics and consultation parameters of all urologic consults from November 2019 to February 2020

Characteristics	Number (n)	Percentage (%=)
Age (Mean)		
<18	04	0.7%
18-30 years	59	10.38%
31-50 years	116	20.42%
51-65 years	144	25.35%
>65 years	245	43.13%
Gender		
Male	418	74%
Female	150	26%
Type of consult		
Urgent	418	74%
Routine (24 hours)	150	26%
Location		
Emergency Department	240	42.25%
Inpatient	318	57.75%
Level of training of Resident		
Level I	237	41.72%
Level II	241	42.42%
Level III-IV	90	15.84%
Time at which consult was raised		
Zone l (00:00-04:00 Hours)	54	9.50%
Zone lI (04:01-08:00 Hours)	45	7.92%
Zone lII (08:01-12:00 Hours)	87	15.31%
Zone lV (12:01-16:00 Hours)	147	25.88%
Zone V (16:01-20:00 Hours)	120	21.12%
Zone Vl (20:01-24:00 Hours)	115	20.24%
Specialty-wise consults		
Emergency Room	240	42.25%
Internal Medicine	158	27.81%
General Surgery	35	6.16%
Nephrology	17	3.0%
Ob/Gyn	17	3.0%
Infectious Diseases	14	2.46%
Cardiology	13	2.28%
Oncology	11	1.93%
Orthopedics	10	1.76%
Pulmonology	9	1.58%
Neurology	9	1.58%
Neurosurgery	8	1.40%
Gastroenterology	6	1.05%
Hematology	5	0.88%
ENT (Ear, Nose & Throat)	5	0.88%
Palliative care	3	0.5%
CTS (Cardio-thoracic surgery)	3	0.5%
Plastic Surgery	2	0.35%
Pediatric Surgery	2	0.35%
Psychiatry	1	0.17%

The most common reason for consultation was hematuria (n=103, 18.13%) followed by obstructive uropathy (n=98, 17.25%). The third most common reason was painful urinary retention (n=53, 9.33%), followed by less common causes which are described in Table 2.

**Table 2:** Reasons for consultation LUTS: lower urinary tract symptoms, UTI: urinary tract infection, UB: urinary bladder

Reason for Consult	Consults (n=)	Percentage (%=)
Hematuria	103	18.13
Obstructive uropathy	98	17.25
Painful acute retention	53	9.33
LUTS/Prostatomegaly	47	8.27
Difficult catheterization	43	7.57
Renal colic	27	4.75
Scrotal wall swelling	25	4.40
Recurrent UTI	24	4.22
Pyelonephritis	22	3.87
Urinary incontinence	16	2.81
Urology on board for ureteric stenting	15	2.64
Renal mass	11	1.93
Suprapubic catheter-related concerns	10	1.76
Nephrostomy-related concerns	7	1.23
Fournier's gangrene	6	1.05
Catheter pull out	5	0.88
Urology on board for UB separation	5	0.88
Prostatitis	4	0.70
Bladder growth	3	0.52
Renal trauma	1	0.17
Miscellaneous	32	5.63

For ease of data analysis, we divided the residents into three levels based on their year of training: Level I comprised Interns, Medical Officers, and PGY I and II residents; Level II included PGY III and IV residents, and Level III included PGY V and VI residents. A vast majority of the consults were seen by junior residents (Level I & II) (n=478, 84.15%), followed by the senior residents, as shown in Table 1.

**Figure 1:**
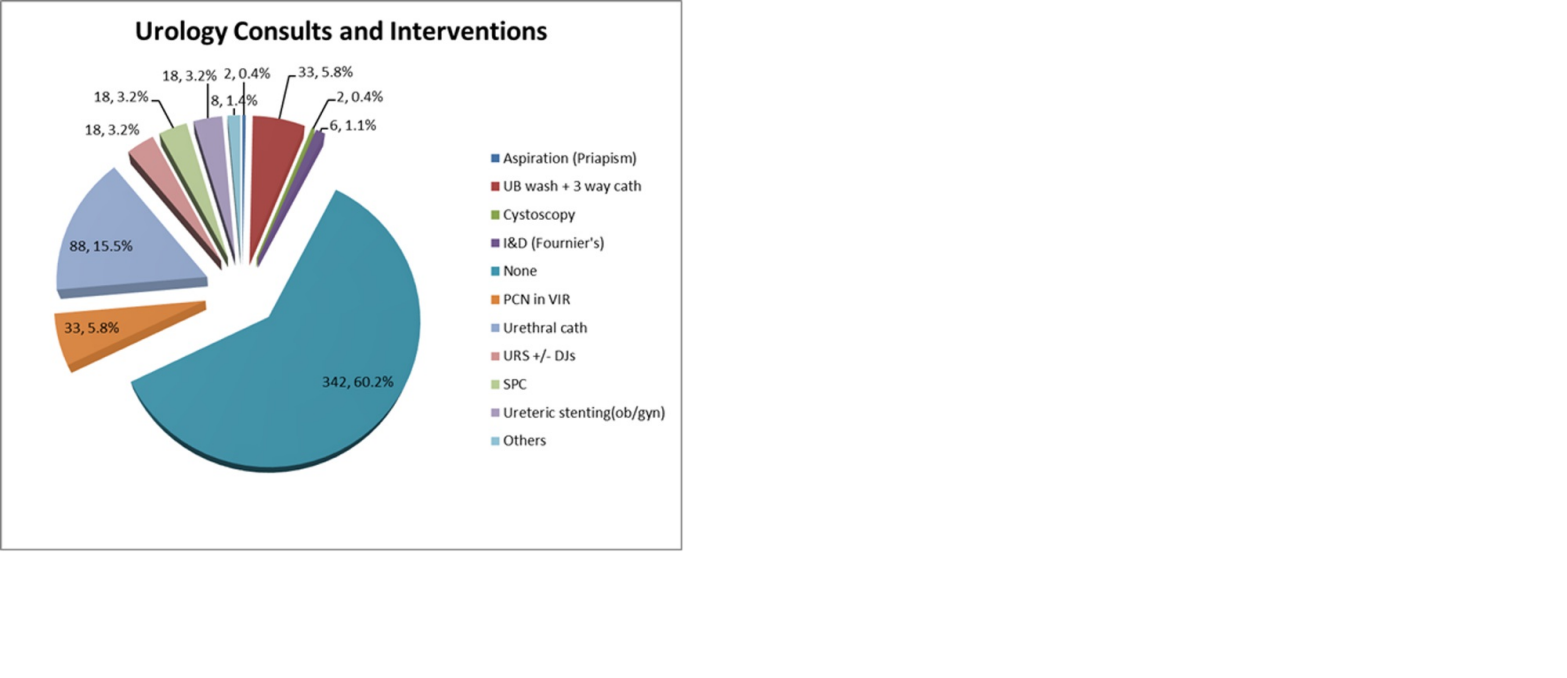
Urology consults and intervention UB: urinary bladder, I&D: incision and drainage, PCN: percutaneous nephrostomy, VIR: interventional radiology, URS: ureterorenoscopy, DJs: double-J stent, SPC: suprapubic catheter

The most common intervention was difficult catheterization (n=88, 15.5%), followed by percutaneous nephrostomy placement for obstruction and bladder wash for hematuria/clot retention (n=33, 5.81%). Less common urologic interventions included: ureterorenoscopy, ureteric stenting and suprapubic catheterization (n=18, 3.17%), incision and drainage (n=6, 1.06%), cystoscopy and aspiration of priapism (n=2, 0.35%), and other interventions (n=8, 1.40%) as shown in Figure 1. Of all the consultation requests, 342 (60.2%) required no urologic intervention and were managed conservatively.

## Discussion

Most large tertiary care hospitals have need for 24-hour in-house urology on-call services [1]. Subspecialty consultations are an integral part of such holistic care as they allow the primary care provider to seek assistance from other colleagues, aiding with the patient’s multi-disciplinary care. Various acute and chronic urologic issues are widely prevalent in the ever-aging population, hence contributing significantly to the health care burden [3,4]. Urological training, particularly in the developing world, is marred by inconsistency and a lack of structure and focus on evidence-based practice. It is believed that improved education and training have the potential to lead to high-quality urological care, and to develop a service that is patient-focused [5].

Little data is available on the prevalence of urologic consultation requests [4]. For the urology trainees, a consult is an “unplanned” clinical activity. Nevertheless, they are indispensable for patients with acute urologic problems. Sparse literature is available highlighting the importance of this “hidden” workload of a urology resident, which can have an effect on their performance [6,7]. Urologists must be accessible since they cover multiple sites, and urgent versus emergent services are required on a nearly daily basis. If this accessibility is limited, then hospital discharge could be delayed unnecessarily [8].

Our study can be a benchmark, describing the incidence and other details of these unplanned clinical activities. In this study, we reviewed the characteristics of inpatient as well as emergency urology consults at a large tertiary care center in Pakistan. We looked at the timing, location, primary service, patient demographics (age and sex), different reasons for consults, and the need for intervention. The four most common reasons for urologic consults were hematuria, obstructive uropathy, painful acute urinary retention, and lower urinary tract symptoms. In addition, our findings suggest that emergency physicians and medical internists are the main specialists frequently seeking urologic input.

The most common interventions performed in our study population included difficult urethral catheterization, percutaneous nephrostomy placement for obstructive uropathy, or bladder wash for hematuria. The fresh residents are often uncomfortable with urethral catheterization, and despite their limited hands-on training, they are still expected to go ahead and perform, as illustrated by Thomas et al. They suggest that basic training to boost competence and confidence with this simple procedure could reduce the morbidity of urethral catheterization [2,9]. Findings from our study correlate with these findings. Most consultation requests are initially seen by the junior team members, who invariably require assistance from the senior residents for simple bedside procedures. We suggest that basic awareness and education needs to be provided to the medical internists about these routine issues.

In a similar study conducted at a tertiary care center in Boston, USA, 857 encounters were analyzed. Urgent encounters involved 19% of patients; 81% were elective. Stones, infection, and urinary retention were the most common diagnoses. One hundred eighty (21%) required patient contact, while 677 (79%) were managed over the telephone. Procedures were needed in 63 (35%) encounters: bladder catheterization in 27 (43%), transurethral surgery in 20 (32%), and ureteroscopy in 16 (25%) [3].

The other end of the spectrum is the frequency of inappropriate and potentially unnecessary consults which result in delayed hospital discharge and a burden for the trainee urologist and the entire service [10]. Patients with non-emergent issues like incidental renal masses, microscopic hematuria, or long-standing lower urinary tract symptoms can be better evaluated in the outpatient setting.

In our series, 60% of consults did not result in any operative or bedside intervention including but not limited to catheterization (suprapubic versus urethral), change of catheter, or bladder wash. Similarly, Sullivan et al. reviewed 711 urology consults at a tertiary referral center in Ireland [6]. In their series, less than half (47%) of the consults required any intervention and half of the interventions were related to catheter issues. We recognize that while the true necessity of a consultation is subjective, the need for urologic intervention is a more quantifiable surrogate. Even then, many interventions do not require urologic expertise [11].

## Conclusions

Our study will be instrumental in the development of a holistic training program for the newly inducted urology residents. It describes urologic consults encountered on a routine basis, and may help devise a generic management plan for each encounter (bladder wash for bladder clots and how to do it, suprapubic catheterization for difficult/failed per-urethral attempts, how to deal with obstructed renal stones, and so on). Although this is a single-center study, the medical conditions are generalizable to most large multidisciplinary hospitals. It also adds to the sparse literature available on the subject.

Based on the findings of our study, a curriculum can be developed for the new urology residents so they may be guided about how to approach every possible scenario as part of the urology on-call team with the maximum exposure to consultation requests.
